# Transfusion of red blood cells does not increase transcutaneous oxygen tension

**DOI:** 10.1186/cc9843

**Published:** 2011-03-11

**Authors:** O Schlager, M Gschwandtner, M Nikfardjam, R Koppensteiner, G Heinz

**Affiliations:** 1 Medical University Vienna, Austria

## Introduction

We investigated the skin oxygen tension (tcpO_2_) of critically ill patients before, during and after transfusion (XF) of packed red blood cells (RBC).

## Methods

Nineteen critically ill patients (11 men, age 67 ± 15 years, SAPS II 60.1 ± 19) who received 2 U RBC due to hemoglobin (Hb) <8 g/l underwent measurement of tcpO_2 _(TCM400; Radiometer Ltd, Copenhagen, Denmark) at the dorsum of one hand. Each patient served as her/his own control (baseline, after XF of 1, and second RBC). Ventilation and pressors were kept constant. Patients with bleeding, in shock and with circulatory assists were excluded. Cardiac index (CI) was determined by FloTrac™/Vigileo™.

## Results

**Figure 1:**
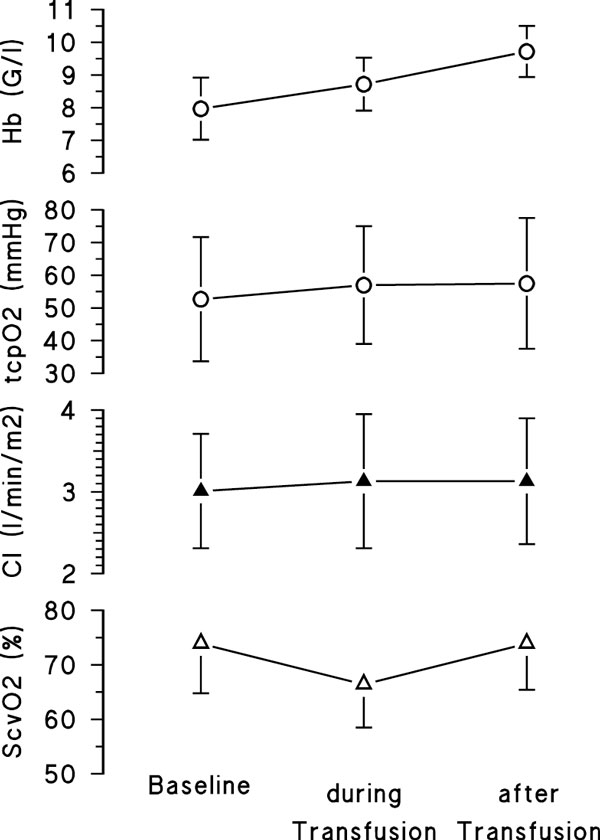


Hb significantly increased (*P *< 0.002), while tcpO_2 _was not significantly different throughout XF (Figure 1; *P *= 0.72). Arterial pO_2 _(86 ± 14 vs. 91 ± 11 vs. 88 ± 18 mmHg, *P *= 0.68) and global hemodynamics (CI, *P *= 0.89, Figure 1; SVR: 822 ± 360 vs. 703 ± 233 vs. 941 ± 410, *P *= 0.13) did not change. Oxygen delivery (DO_2_) significantly increased (644 ± 188 vs. 744 ± 234 vs. 818 ± 214 ml/minute, *P *= 0.049). Interestingly, central venous oxygen saturation (ScvO_2_) decreased significantly during XF and did not completely recover until the end of XF (*P *< 0.05 midst XF vs. baseline; Figure 1).

## Conclusions

XF significantly increased Hb and calculated DO_2 _but not true tcpO_2_. Increase in DO_2 _occurred in the absence of changes in CI and oxygenation. ScvO_2 _significantly decreased during XF but did not completely recover until the end of the study period.

